# Differential association of antioxidative defense genes with white matter integrity in youth bipolar disorder

**DOI:** 10.1038/s41398-022-02261-w

**Published:** 2022-12-07

**Authors:** Yi Zou, Anahit Grigorian, Kody G. Kennedy, Clement C. Zai, Suyi Shao, James L. Kennedy, Ana C. Andreazza, Stephanie H. Ameis, Chinthaka Heyn, Bradley J. Maclntosh, Benjamin I. Goldstein

**Affiliations:** 1 Department of Pharmacology, University of Toronto, Toronto, ON Canada; 2 Centre for Youth Bipolar Disorder, Centre for Addiction and Mental Health, Toronto, ON Canada; 3 Psychiatric Neurogenetics Section, Tanenbaum Centre for Pharmacogenetics, Molecular Brain Science Department, Campbell Family Mental Health Research Institute, Centre for Addiction and Mental Health, Toronto, ON Canada; 4 Department of Psychiatry, University of Toronto, Toronto, ON M5T 1R8 Canada; 5 Cundill Centre for Child and Youth Depression, Margaret and Wallace McCain Centre for Child, Youth & Family Mental Health, Campbell Family Mental Health Research Institute, Centre for Addiction and Mental Health, Toronto, Canada; 6 Department of Psychiatry, Temerty Faculty of Medicine, University of Toronto, Toronto, ON Canada; 7 Department of Psychiatry, The Hospital for Sick Children, Toronto, ON Canada; 8 Department of Medical Imaging, Sunnybrook Health Sciences Centre, Toronto, ON Canada; 9 Heart and Stroke Foundation, Canadian Partnership for Stroke Recovery, Sunnybrook Research Institute, Toronto, ON Canada; 10 Department of Medical Biophysics, University of Toronto, Toronto, ON Canada; 11 Hurvitz Brain Sciences Program, Sunnybrook Research Institute, Toronto, ON Canada

**Keywords:** Bipolar disorder, Clinical genetics

## Abstract

Oxidative stress is associated with white matter diffusion metrics in adults with bipolar disorder (BD). We examined the association of single-nucleotide polymorphisms in the oxidative stress system, superoxide dismutase-2 (*SOD2*) rs4880 and glutathione peroxidase-3 (*GPX3*) rs3792797 with fractional anisotropy (FA) and radial diffusivity (RD) in youth with BD. Participants included 104 youth (age 17.5 ± 1.7 years; 58 BD, 46 healthy controls). Saliva samples were obtained for genotyping, and diffusion tensor imaging was acquired. Voxel-wise whole-brain white matter diffusion analyses controlled for age, sex, and race. There were significant diagnosis-by-*SOD2* rs4880 interaction effects for FA and RD in major white matter tracts. Within BD, the group with two copies of the G-allele (GG) showed lower FA and higher RD than A-allele carriers. Whereas within the control group, the GG group showed higher FA and lower RD than A-allele carriers. Additionally, FA was higher and RD was lower within the control GG group compared to the BD GG group. No significant findings were observed for *GPX3* rs3793797. The current study revealed that, within matter tracts known to differ in BD, associations of *SOD2* rs4880 GG genotype with both FA and RD differed between BD vs healthy control youth. The SOD2 enzyme encoded by the G-allele, has higher antioxidant capacity than the enzyme encoded by the A-allele. We speculate that the current findings of lower FA and higher RD of the BD GG group compared to the other groups reflects attenuation of the salutary antioxidant effects of GG genotype on white matter integrity in youth with BD, in part due to predisposition to oxidative stress. Future studies incorporating other genetic markers and oxidative stress biomarkers are warranted.

## Introduction

Bipolar disorder (BD) is a highly heritable, polygenic psychiatric disorder with an early onset, often during youth and young adulthood [1, 2]. There is robust evidence of structural and functional white matter (WM) anomalies in BD [3, 4]. Diffusion tensor imaging (DTI), a magnetic resonance imaging technique that is sensitive to the movement of water in biological tissue, provides a non-invasive proxy measurement of WM tissue microstructure [5]. Fractional anisotropy (FA) reflects the movement of water molecules, where a higher value (range = 0–1) is indicative of preferential diffusion in one direction relative to more restricted diffusion in another orthogonal direction [6, 7]. In contrast, radial diffusivity (RD) measures diffusivity that is perpendicular to the main diffusion direction [7, 8]. There is replicated evidence of reduced FA and increased RD across major WM tracts in both youth and adults with BD, as well as relatives of individuals with BD, compared to controls [9–18]. FA is thought to reflect various WM microstructural changes including myelin anomalies, reduction in white matter density, and a loss in fiber bundle coherence, and RD is thought to reflect myelin integrity [19–21]. When reduced FA is observed in parallel with increased RD, this is thought to reflect myelin sheet damage/dysmyelination [7, 8]. Despite this evidence, little is known regarding the putative biological mechanisms underlying anomalous FA and RD changes in BD.

Oxidative stress, defined as the imbalance between oxidant generation and antioxidative capacity, is one of the leading etiopathological mechanisms in BD [22], encompassing findings from both peripheral biomarker and post-mortem brain studies [23–26]. The brain has high metabolic demands, with low antioxidative capacity and high levels of lipids [27, 28]. These properties render the brain particularly susceptible to oxidative damage [29, 30]. The antioxidative enzymes such as catalase, superoxide dismutase (SOD), and glutathione peroxidase (GPX) are encoded in the human genome and play an essential role in helping to eliminate reactive oxygen species (ROS).

The current study focuses on superoxide dismutase-2 (*SOD2*) rs4880 and glutathione peroxidase-3 (*GPX3*) rs3792797, single nucleotide polymorphisms (SNPs) for genes coding for the two major antioxidant enzymes. Prior genetic studies in youth and adults have shown that these SNPs are associated with BD [31, 32]. The SOD2 enzyme acts mainly in the mitochondria. The rs4880 SNP (Ala16Val) results in a structural change, which increases its ability to cross the mitochondrial double membrane, and a functional change that increases it’s enzymatic activity [33–36]. The GPX3 enzyme is a major extracellular antioxidant enzyme [37]. One prior study reported a higher GPX3 gene expression with increasing age in a healthy population, suggesting a neuroprotective effect of GPX3 during brain development [38].

The rich lipid content of myelin tracts render them especially susceptible to oxidative stress damage [39]. Relatedly, prior studies have found that oxidative stress is associated with changes in FA and RD [29, 40, 41]. A study of elderly adults examined whether the *SOD2* rs4880 polymorphism was associated with WM integrity, finding that the GG homozygotes, individuals who carry two copies of the G-allele, had higher RD in the anterior thalamic radiation as compared to A-allele carriers [40]. Another study examining the association between brain glutathione levels and FA in the cortical-limbic circuit in a population sample of young adults with unipolar depression (*n* = 94) or BD (*n* = 76) revealed that among those with low glutathione, lower levels of this antioxidant were associated with lower FA [41]. Finally, a study of adults with BD found that higher lipid peroxidation was associated with lower FA, and lipid peroxidation markers explained 59% of the variance in FA [29]. These converging findings suggest a potential role of the anomalous antioxidant defense mechanisms in BD-associated WM anomalies.

To date, no prior studies have examined the association between the *SOD2* rs4880 or *GPX3* rs3792797 in relation to WM anomalies in youth or in BD. The current study aims to explore the association of these SNPs with FA and RD in youth with vs. without BD using a whole-brain analysis approach. We hypothesized that there would be differential associations of genotype with DTI metrics. Given the lack of prior findings, we approached hypothesis-testing agnostic to directionality of effects.

## Methods and Materials

### Participants

One-hundred and four English speaking youth participants between the ages of 13–20 years old were recruited in the current study, with 58 BD and 46 healthy controls (HC). *GPX3* rs3792797 genotype data were missing for two participants, yielding data for 102 participants. For *SOD2* rs4880 SNP, participants were divided into three subgroups: those carrying two copies of the A-allele (AA: BD = 12; HC = 13), those carrying two copies of the G-allele (GG: BD = 21; HC = 12), and those carrying one copy of the A-allele and one copy of the G-allele (AG: BD = 25; HC = 21). For *GPX3* rs3792797, participants were divided into two groups: those carrying one or two copies of the A-allele (A-allele carriers [AA or AC]: BD = 17; HC = 20), and those carrying two copies of the C-allele (CC: BD = 41; HC = 24). Written informed consent was obtained from all participants and one of their guardian(s) prior to participation. HC were recruited via hospital and community advertisements, with the following exclusion criteria: lifetime mood or psychotic disorders; family history of BD and/or other psychotic disorder; exposure to psychiatric medications or substance dependence or anxiety disorder in the past three months. BD participants with diagnosis of BD-I (bipolar I disorder), BD-II (bipolar II disorder), or BD-NOS (bipolar-not other specified) were recruited from a tertiary subspecialty outpatient clinic. Exclusion criteria were: having cardiac, autoimmune, or inflammatory conditions; taking hyperglycemic, anti-hypertensive, anti-platelet, anti-lipidemic, or daily anti-inflammatory medications; having substance dependence in the past three months; or having contraindications to magnetic resonance imaging (MRI). The study was approved by the Sunnybrook Research Institute Research Ethics Board (REB 408–2011, 409–2013). The study was also approved by the by the Centre for Addiction and Mental Health (CAMH) Research Ethics Board (REB 165/2020, 168/2020), as data analyses were undertaken at CAMH following the Centre for Youth Bipolar Disorder’s relocation to CAMH.

### Psychiatric and anthropometric measures

The Kiddie Schedule for Affective Disorders and Schizophrenia for School-Age Children, Present and Lifetime version (KSADS-PL) semi-structured diagnostic interview was employed for psychiatric diagnoses, and clinical/demographic data collection [42]. Since participant recruitment occurred between 2012 to 2019, the Diagnosis and Statistical Manual of Mental Disorders, fourth edition (DMS-IV) was used for psychiatric diagnoses, as the DSM5 version of KSADS was not available until 2016 [42]. Diagnosis and symptom severity were evaluated using the KSADS Depression Rating Scale and the Mania Rating Scale [43, 44]. Given the lack of operationalized criteria in DSM-IV, BD-NOS was defined based on the COBY(Course and Outcome of Bipolar Illness in Youth) study [45]. Diagnoses were reviewed in consensus conferences with a licensed child-adolescent psychiatrist. Clinical study data was collected and managed using REDCap, (Research Electronic Data Capture) a secure web-based software platform designed to support data capture for research studies. Age of BD onset was defined as the age at which the participant first experienced an episode of mania or hypomania, or met diagnosis criteria for BD-NOS. Self-reported race was recorded. Family psychiatric history was collected from first- and second-degree relatives using the Family History Screen Interview [46]. As part of the K-SADS-PL interview, information regarding psychotropic medication use and tobacco use were collected as well. Pubertal stage was evaluated using the Pubertal Developmental Scale [47]. All interviewers were trained by the senior author.

Participant’s height (cm) and weight (kg) were collected twice at intake. Values were then averaged according to the standard procedure for both weight and height for precision [48]. Body mass index (BMI) was calculated as weight in kilograms over height in meters squared.

### DNA extraction and genotyping

Genetic data were obtained from genomic DNA purified from 2 mL saliva samples (DNA Genotek Oragene-500 kits; DNA Genotek Inc., Ottawa, Canada) for each participant. Instructions were provided to ensure participants abstained from eating, drinking, smoking/vaping and chewing gum 30 min prior to saliva sample collection. DNA extraction and genotyping were performed by the CAMH Biobank and Molecular Core Facility. DNA extraction using the chemagicTM MSM I DNA extractor (Perkin-Elmer, Waltham, MA) was followed by quantification and quality assessment using Nanodrop 8000 spectrophotometer (ThermoFisher Scientific, Waltham, MA). The sample was diluted to a final concentration of 20 ng/µL. Genotyping was then performed for the *SOD2* rs4880 and *GPX3* rs3792797 SNPs using TaqMan® Format 32 OpenArray® Genotyping Plates (ThermoFisher Scientific, Waltham, MA) as per manufacturer’s instructions, with amplification being carried out in the QuantStudio™ 12 K Flex Real-Time PCR System (ThermoFisher Scientific). The genotype data were imported and reviewed by two-independent researchers using the TaqMan® Genotyper software version 1.3. All technicians were blind to diagnosis. PLINK software version 1.90 was used to examine potential sampling bias with the Hardy-Weinberg equilibrium analysis. No significant deviation from Hardy-Weinberg equilibrium was observed (*p* > 0.05) [49, 50]. For *SOD2* rs4880 and *GPX3* rs3792797 the allele frequencies are reported in Table 1 for the current study sample and for reference groups. For *SOD2* rs4880, the A-allele frequency were 0.42 for BD, 0.51 for CG, 0.46 for the overall sample, and 0.5 for the European reference sample. For GPX3 rs3792797, the A-allele frequency were 0.16 for BD, 0.3 for CG, 0.22 for the overall sample, and 0.18 for the European reference sample. Overall, allele frequencies observed in the current sample are aligned with those observed in the reference samples.

**Table 1:** Allele frequencies for *SOD2* rs4880 and *GPX3* rs3792797.

		*SOD2* rs4880		*GPX3* rs3792797	
		A-allele	G-allele	A-allele	C-allele
Current Sample	BD	0.42	0.58	0.16	0.84
	HC	0.51	0.49	0.30	0.70
	Overall Sample	0.46	0.54	0.22	0.78
Reference Sample	BD European Descent [75]	0.50	0.50	0.18	0.82
	Global	0.51	0.49	0.20	0.80
	European	0.50	0.50	0.18	0.82

*BD* Bipolar disorder, *HC* Healthy control. BD European Descent allele frequencies was based on Mullins et al., 2021 publication. Global and European allele frequencies was based on the National Center for Biotechnology Information (NCBI) SNP database. Allele frequencies were calculated based on the following equation: *p*² + 2*pq* + *q*² = 1.

### Imaging acquisition

Diffusion-weighted data were acquired using a single-shot, spin-echo planar imaging (EPI) sequence on a 3 Tesla Philips Achieva MRI scanner (Philips Medical Systems, Best, Netherlands). For each participant, diffusion data were collected along 32 gradient directions at a *b* value of 1000 s/mm^2^. Seven images with no diffusion weighting were obtained. The acquisition parameters were as follow: TR/TE = 9150/55 ms; flip angle=90; field-of-view [FOV] = 224×224; fifty-two 3mm-thick slices; matrix size=128×128; acquisition duration: 6’27”. The axial imaging plane was prescribed obliquely to align with the anterior-to-posterior commissure.

### DTI image processing

Diffusion data processing and analysis were performed using FSL FMRIB Software Library tools (FMRIB, Oxford Center for Functional MRI of the Brain, University of Oxford) [51]. Diffusion-weighted images were eddy-current corrected and brain extracted prior to tensor fitting using DTIFIT to calculate DTI metrics. Individual fractional anisotropy (FA) and radial diffusivity (RD) maps were computed from the tensor eigenvalues (λ1, λ2, λ3). Tract-based spatial statistics (TBSS), part of FSL, was carried out to align each participants’ major WM tracts with a mean FA skeleton for voxel-wise statistics. Specifically, all participants’ FA maps were registered to a study-specific target chosen from the adolescent sample, and then transformed into MNI space. A skeleton of the mean of all FA maps in standard space was then calculated, and a threshold of 0.3 was applied to exclude non-WM voxels and to remove the high inter-subject variability at tract extremities [18, 52]. Finally, a group skeletonized 4D FA image was generated and used as the input for voxel-wise analysis with randomise. The same steps were applied to participants’ RD maps. Images were inspected for quality after each step by two independent raters.

### Statistical analyses

Clinical and demographic group differences were evaluated using the SPSS statistic software (IBM; NY, USA), version 26. Normality and equal variance assumptions of all continuous variables were examined using Shapiro-Wilks test and Levene’s test, respectively, prior to conducting the group analyses. Two-way analysis of variance (ANOVA) and chi-square (χ2) tests were performed to evaluate group differences for continuous and categorical variables.

**Fig. 1:**
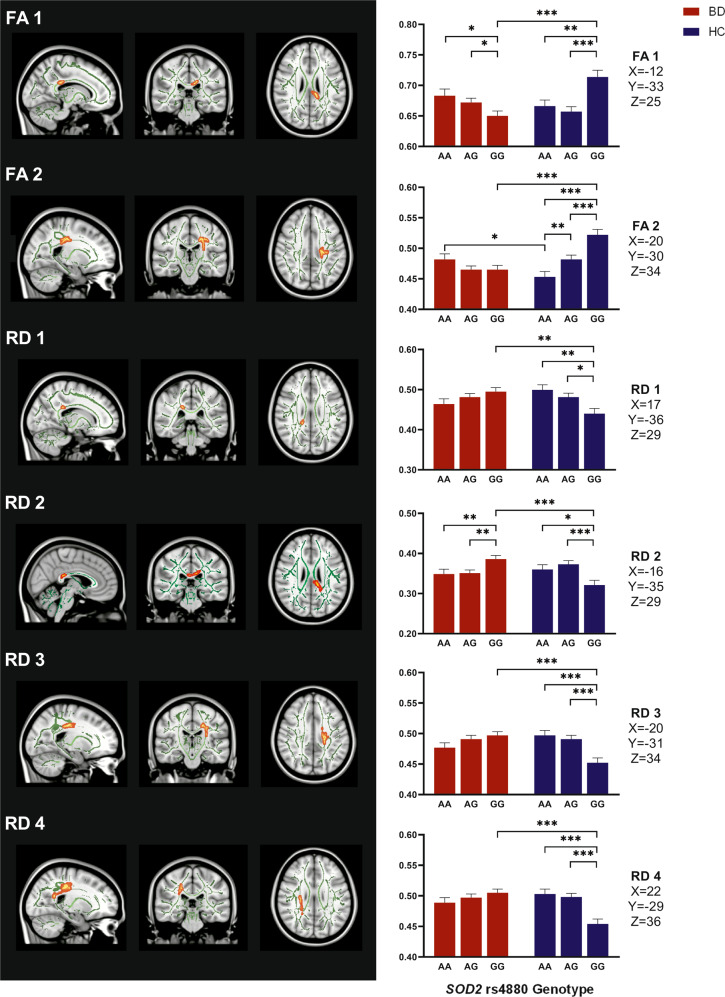
Post hoc analyses for clusters with significant diagnosis-by-genotype interaction effects. *p < 0.05, **p < 0.01, ***p < 0.001. FA1)-FA2) Diagnosis-by-*SOD2* rs4880 interaction effect post-hoc results for significant FA clusters. FA1 cluster peaks in the splenium of the corpus callosum and FA2 cluster peaks in the posterior corona radiata and anterior thalamic radiation. RD1)-RD4) Diagnosis-by-*SOD2* rs4880 interaction effect post-hoc results for significant RD clusters. RD1 peaks in the right and RD2 peaks in the left splenium of corpus callosum. RD3 peaks in the left and RD4 peaks in the right posterior corona radiata. Information regarding the peak anatomical location for each cluster was reported as unit of mm in the X, Y, and Z directions.

Main effects and interaction effects: Whole brain voxel-wise contrasts for diagnosis and genotype main effects, and diagnosis-by-genotype interaction effects for each DTI metric (FA and RD) were performed, separately for *SOD2* and *GPX3* using a General Linear Model (GLM) with age, sex, and race as covariates. Analyses was performed using FSL randomize tool with 5000 permutations. Significance level was set at *p* < 0.05 and results were corrected for multiple comparisons using the family-wise error rate (FWER) correction with the Threshold-Free Cluster Enhancement (TFCE) thresholding option. FSL Cluster tool was used to obtain cluster size, anatomical coordinates, and peak *p* value of significant clusters. The corresponding white matter tract was identified using the CBM-DTI-81 white-matter labels or the JHU White-Matter Tractography atlases. Lastly, for better visualization purposes, significant clusters (Fig. 1) were thickened using the FSL tool *tbss_fill*. Sensitivity analyses were conducted further covarying for lithium and second-generation antipsychotics (SGA) independently in the statistical model.

Post-hoc pairwise contrasts: Post-hoc analyses were conducted, using MATLAB version R2019b, to follow-up on significant findings (main effect and/or interaction effect) in order to reveal directionality. Bonferroni correction was used to correct for multiple post-hoc pairwise comparisons (*SOD2* SNP gene main effect: α = 0.05/3 = 0.016; *SOD2* SNP diagnosis-by-genotype interaction effect: α = 0.05/9 = 0.005) for the post-hoc analyses.

## Results

### Demographics and clinical characteristics

Demographic characteristics are summarized in Table 2. There were no significant demographic differences between diagnostic groups. For the *SOD2* polymorphism, BMI was higher for the AG genotype group compared to the AA genotype group (*p* = 0.03) and Tanner stage was higher in the GG group compared to the AA group (*p* = 0.03). No difference was observed for age, sex, and race. For the *GPX3* polymorphism, age was higher in the A carrier group as compared to the CC homozygous group (*p* = 0.047). No difference was observed for sex, race, BMI, and Tanner stage. Clinical characteristics for the BD group are summarized in Table 3.

**Table 2:** Demographic Characteristics by Diagnosis and SNPs in two antioxidant enzyme genes.

	Diagnosis				
	BD (*n* = 58)	HC (*n* = 46)	F/*χ*^2^	*p*	η_p_^2^*/V*
Age	17.7 ± 0.2	17.2 ± 0.3	2.26	0.14	0.02
Sex (*n*, % female)	38 (66%)	23 (50%)	2.55	0.11	0.16^*V*^
Race (*n*, % Caucasian)	41 (71%)	24 (52%)	3.75	0.053	0.19^*V*^
BMI (adjusted)	24.0 ± 0.6	22.5 ± 0.7	2.74	0.10	0.03
Tanner Stage	4.5 ± 0.1	4.2 ± 0.1	2.93	0.09	0.03

	*SOD2* rs4880					
	AA (*n* = 25)	AG *(n* = 46)	GG (*n* = 33)	F/*χ*^2^	*p*	η_p_^2^*/V*
Age	17.6 ± 0.3	17.5 ± 0.3	17.3 ± 0.3	0.19	0.82	0.004
Sex (*n*, % female)	12 (48%)	26 (57%)	23 (70%)	2.92	0.23	0.17^*V*^
Race (*n*, % Caucasian)	13 (52%)	29 (63%)	23 (70%)	1.91	0.39	0.14^*V*^
BMI (adjusted)^a^	22.1 ± 0.9	24.9 ± 0.6	22.8 ± 0.8	3.74	**0.03**	0.07
Tanner Stage^a^	4.1 ± 0.1	4.3 ± 0.1	4.6 ± 0.1	3.54	**0.03**	0.07

	*GPX3* rs3792797				
	CC (*n* = 65)	AA/AC* (*n* = 37)	F/*χ*^2^	*p*	η_p_^2^*/V*
Age^a^	17.1 ± 0.2	17.8 ± 0.3	4.05	**0.047**	0.04
Sex (*n*, % female)	40 (62%)	19 (51%)	1.00	0.32	0.10^*V*^
Race (*n*, % Caucasian)	42 (65%)	23 (62%)	0.06	0.80	0.03^*V*^
BMI (adjusted)	24.0 ± 0.6	22.9 ± 0.7	1.31	0.25	0.01
Tanner Stage	4.3 ± 0.1	4.4 ± 0.1	0.15	0.70	0.002

Results are reported in mean ± standard deviation (*SD*) unless otherwise specified. *BD* Bipolar disorder, *HC* Healthy control, *BMI* Body mass index. Significant differences are Bolded.

*AA/AC combined due to limited subgroup size.

^a^Indicates significant differences between genotype groups.

Missing cases (n): Tanner Stage (1).

Effect size was reported in as partial eta squared=η_p_^2^ for F-test and Cramér’s V = *V* for Crosstab.

**Table 3:** Clinical characteristics.

	BD (*n* = 58)
BD-I	25 (43%)
BD-II	17 (29%)
BD-NOS	16 (28%)
Age of BD Onset	14.97 ± 2.68
**Lifetime Clinical Characteristics**	
Lifetime Psychosis	13 (22%)
Lifetime Suicide Attempts	8 (14%)
Lifetime Self-injurious Behaviour	27 (47%)
Lifetime Suicidal Ideation	33 (57%)
Lifetime Any Abuse (physical and/or sexual)	6 (10%)
Lifetime Psychiatric Hospitalization	28 (48%)
**Lifetime Comorbid Diagnoses**	
ADHD	28 (48%)
Any Anxiety Disorder	47 (81%)
Number of Anxiety Disorders	1.90 ± 1.36
Conduct Disorder	2 (3%)
Oppositional Defiant Disorder	17 (29%)
Substance Use Disorder	14 (25%)
Nicotine Use	8 (14%)
**Lifetime Medications**	
Second generation antipsychotics	45 (78%)
Lithium	14 (24%)
Non-SSRI Antidepressants	9 (16%)
SSRI Antidepressant	20 (35%)
Stimulants	17 (29%)
**Current Medications**	
Second Generation Antipsychotics	31 (53%)
Lithium	11 (19%)
Non-SSRI Antidepressants	1 (2%)
SSRI Antidepressants	9 (16%)
Stimulants	7 (12%)
**Family Psychiatric History**	
Mania/Hypomania	34 (59%)
Depression	45 (78%)
Anxiety	30 (52%)
SUD	12 (21%)
Psychosis	14 (25%)
ADHD	20 (35%)

*BD* Bipolar disorder, *NOS* Not otherwise specified, *HC* Healthy control, *ADHD* Attention deficit-hyperactivity disorder, *SSRI* Selective serotonin reuptake inhibitor, *SUD* Substance use disorder. Note: Results are reported in mean ± standard deviation (SD) or percentage (%) Yes unless otherwise specified.

Missing cases (n): Family Psychiatric History Psychosis (2).

### SOD2 rs4880

Voxel-wise whole brain analyses for *SOD2* polymorphism were summarized in Table 4. There were two clusters with a significant genotype main effect for the FA metric (Supplementary Table 1). Findings remained significant in sensitivity analyses further controlling for current lithium or SGA use. One cluster had peaks in the anterior thalamic radiation and superior corona radiata (*p* = 0.04), with significantly lower FA in the AA and AG groups vs the GG group. A second cluster had two peaks in the right corticospinal tract (*p* = 0.04), with significantly lower FA in the AG group as compared to the AA and GG groups.

**Table 4:** Results for whole brain analyses.

SNPs	Contrast	Metrics	Cluster Number	Voxel Numbers	*p*Max	Cluster Peak			
						Anatomical Location	X (mm)	Y (mm)	Z (mm)
SOD2	Gene main effect	FA	Cluster 1	51	0.04	Anterior thalamic radiation R Superior corona radiata R	23	−21	36
			Cluster 2	72	0.04	Corticospinal tract R	18	−6	44
	Interaction effect	FA	Cluster 1	119	0.03	Splenium of corpus callosum L	−12	−33	25
			Cluster 2	632	0.006	Posterior corona radiata L Anterior thalamic radiation L	−20	−30	34
		RD	Cluster 1	30	0.048	Splenium of corpus callosum R	17	−36	29
			Cluster 2	321	0.04	Splenium of corpus callosum L	−16	−35	29
			Cluster 3	436	0.048	Posterior corona radiata L	−20	−31	34
			Cluster 4	716	0.021	Posterior corona radiata R	22	−29	36
GPX3	Gene main effect	FA	Cluster 1	102	0.037	Retrolenticular part of internal capsule R Inferior fronto-occipital fasciculus R Inferior longitudinal fasciculus R	34	−33	8

*FA* fractional anisotropy, *RD* radial diffusivity, *SNP* single nucleotide polymorphism.

There were significant diagnosis-by-genotype interaction effects for FA in two clusters: one (Fig. 1, FA1) with a peak in the splenium of the corpus callosum and another (Fig. 1, FA2) with peaks in the posterior corona radiata and anterior thalamic radiation (Table 4). Findings remained significant in sensitivity analyses further controlling for current lithium or SGA use. Post-hoc analyses revealed higher FA in the HC GG group as compared to the BD GG, HC AA and AG groups for both FA clusters (Supplementary Table 2). For FA cluster #1 the BD GG group revealed significantly lower FA as compared to the BD AG and BD AA groups. These within BD group differences did not remain significant after Bonferroni correction for multiple comparisons. For cluster #2, the HC AA group showed lower FA as compared to the HC AG and BD AA groups. However, this finding did not remain significant after correction for multiple comparisons. For the sensitivity analysis, all post-hoc findings remained significant except for the between BD AG and GG group comparison with SGA as an additional covariate for cluster #1. However, the effect size measured using eta square (η^2^) were similar in direction and magnitude (large effect) for the SGA sensitivity post-hoc analysis (η^2^ = 0.12) as compared to the main analysis post-hoc (η^2^ = 0.28).

There were also significant diagnosis-by-genotype interaction effects for RD in four clusters: where cluster #1 (Fig. 1, RD1) and #2 (Fig. 1, RD2) had peaks in the right (*p* = 0.048) and left (*p* = 0.04) splenium of corpus callosum, respectively, and cluster #3 (Fig. 1, RD3) and #4 (Fig. 1, RD4) had peaks in the left (*p* = 0.048) and right (*p* = 0.02) posterior corona radiata, respectively. All findings remained significant in sensitivity analyses further controlling for current lithium or SGA use. Post-hoc analyses revealed the HC GG group showed significantly lower RD as compared to BD GG, HC AA and HC AG groups for all four clusters (S2). For cluster #2, significant within-BD group differences were observed; RD was significantly higher in the BD GG group compared to the AA and AG genotype groups. However, the within-BD differences between GG and AA groups did not survive correction for multiple comparisons. For the sensitivity analysis, all post-hoc findings remained significant except the cluster #1 post-hoc finding of differences between BD GG and HC GG groups after controlling for current SGA. However, the effect size was of similar magnitude and in the same direction (η^2^ = 0.06) as compared to the main analysis (η^2^ = 0.09).

### GPX3 rs3792797

Results of voxel-wise whole brain analysis for *GPX3* rs3792797 are summarized in Table 4. There was a significant main effect of rs3792797 on FA in a cluster with peaks in the following white matter tract regions: the right retrolenticular part of the internal capsule, the inferior fronto-occipital fasciculus, and the inferior longitudinal fasciculus (*p* = 0.04). Finding remained significant in sensitivity analyses with current lithium or SGA as additional covariates. Post-hoc analyses revealed that the difference was due to lower FA in the CC group (estimated marginal mean=0.536) as compared to the A-carrier group (estimated marginal mean=0.574). There was no significant interaction effect between *GPX3* rs3792797 and diagnosis.

## Discussion

The present study examined the association of SNPs in genes involved in oxidative defense with white matter integrity in youth with and without BD. Whole-brain voxel-wise analyses revealed significant interaction effects between *SOD2* rs4880 and diagnosis in various major white matter regions, including the splenium of corpus callosum, posterior corona radiata, and anterior thalamic radiation. For the identified white matter tracts, post-hoc analyses revealed significantly higher FA and lower RD in the HC GG group as compared to the BD GG group, and other HC genotypes. In addition, within-BD differences were observed for the splenium of the left corpus callosum, with the BD GG group showing lower FA (not significant after multiple comparison correction) and higher RD than the other genotype groups. No significant interaction effect was observed for the *GPX3* rs3792797 SNP. In summary, the current study reveals a significant association of the *SOD2* rs4880 SNP with FA and RD in major white matter tracts previously implicated in BD. Disturbances in oxidative biology and anomalous white matter integrity are well established in BD. Results of the current study suggest that these two well established findings may be related to one another.

The mechanisms underlying the association of the *SOD2* rs4880 SNP with white matter integrity are currently unclear, with mixed reports of a possible protective role versus a potentially deleterious effect on the brain. *SOD2* rs4880 results in the substitution of the amino acid valine (Val) with alanine (Ala) at position 16 [53]; this results in secondary structure changes of the polypeptide, leading to changes in the three-dimensional folding pattern of the antioxidative enzyme [33]. These protein structure changes have been shown to increase the ability of the Ala-encoded SOD2 enzyme to cross the inner mitochondrial membrane, resulting in higher availability of this enzyme inside the matrix [34, 35]. In addition, the Ala-encoded SOD2 enzyme has also been reported to be more enzymatically active compared to the Val-encoded enzyme [54]. One can speculate that the higher availability and activity of the SOD2 enzyme might thus be beneficial in normal circumstances, scavenging superoxide radicals by conversion to hydrogen peroxide (H_2_O_2_), which is in turn eliminated after being converted to H_2_O. In the present study, we observed that the HC GG group has the highest FA and lowest RD compared to the BD GG group and other HC genotype groups. Prior literature has reported high lipid content (~80%) in axonal fibers and myelin sheets, rendering these white matter structures especially susceptible to ROS-related lipid peroxidation [39]. The inverse pattern of FA and RD observed in the HC GG genotype group might suggest that the Ala-encoded SOD2 enzyme may have a protective effect on white matter integrity through enhanced antioxidative capacity [55].

In contrast, the BD GG group was associated with lower FA (not significant after multiple comparison correction) and higher RD than the HC GG group and the other BD genotypes, reflective of oxidative stress-related damage to axonal fibers and myelin sheets [6–8]. Although tentative and preliminary, these divergent findings might suggest that neuroprotective effects of the G allele are lacking in BD, differences that may be explained by the predisposition to redox imbalances in this population [56–58]. Essentially, in circumstances where oxidative stress overwhelms antioxidative capacity, the increased SOD2 enzymatic availability and activity that is associated with the G allele ultimately results in increased generation of H_2_O_2_ with impaired capacity to efficiently convert this ROS to H_2_O [27, 53, 54]. ROS, especially H_2_O_2_, regulates myelination processes [59, 60] and, when produced in excess in oligodendrocytes, induces direct cell death in vitro [61]. In summary, alterations in brain ROS levels may have both a direct impact on axons and myelin integrity via lipid peroxidation, as well as indirect impact via regulation of oligodendrocyte myelin production.

The current study found a differential association of the *SOD2* rs4880 GG genotype with FA and RD in the splenium of corpus callosum and posterior corona radiata. The splenium of corpus callosum is the posterior part of the corpus callosum, consisting of axonal fibers connecting the temporal, posterior parietal, and occipital cortices [62]. A recent meta-analysis examining 57 studies of young and middle-aged adults reported that FA was significantly lower, and RD was significantly higher, in the splenium of corpus callosum in participants with BD compared to HC [63]. In addition, this meta-analysis included studies that examined individuals at familial-genetic risk for BD. Compared to the HC group, the group with increased risk for BD had lower FA in the CC, including the splenium subregion [63]. Similarly, lower FA has been observed in the splenium of corpus callosum in youth with BD compared to HC [16, 64]. For posterior corona radiata, prior studies have reported lower FA [65–67] and higher RD [68] in adults with BD compared to HC; additionally, there is evidence of lower FA among adults at familial-genetic risk for BD compared to HC [69, 70].

We found a significant diagnosis-by-genotype interaction on anterior thalamic radiation FA. This white matter tract travels through the anterior limb of the internal capsule and connects the prefrontal cortex with the thalamus, specifically the dorsomedial thalamic nucleus [71]. Lower FA of the anterior thalamic radiation has been previously reported in adolescents [72] and adults [73] with BD compared to HC. The aforementioned meta-analysis in young and middle-aged adults confirmed reduced FA in this region in BD compared to HC [63]. In addition, lower FA of the anterior thalamic radiation has been previously reported in unaffected relatives of BD patients, aged between 16 to 25 years old, when compared to HC [74].

It is important to acknowledge that present findings are constrained by a number of limitations. First, the cross-sectional design impedes our ability to make inferences regarding the temporal association between the antioxidative enzyme SNPs and white matter integrity. Second, the sample size is limited. For this reason, homozygous and heterozygous *GPX3* rs3792797 A-allele carriers were grouped together. Similarly, the limited sample size precluded evaluation of additive genetic effect analyses or subgroup analyses (i.e., sex differences, age differences, ethnicity, BD subtypes, BD mood states). Finally, additional DTI metrics such as axial diffusivity and mean diffusivity, while potentially informative, were deferred due to concern about multiple contrast in this small sample. In addition, the current sample size, although relatively large for an imaging study in the youth BD population, is not powered to detect small effects. Lastly, we did not include oxidative stress biomarkers (e.g. protein levels, gene expression). Including these measurements might be beneficial for understanding the biological mechanism underlying the antioxidative defense genes with BD-associated anomalous diffusion metrics.

To conclude, the current study provides initial evidence regarding the differential association of the *SOD2* rs4880 SNP with FA and RD in youth with vs. without BD. The observed findings may suggest reduced protective effects of the G allele on axonal and myelin integrity in youth with BD due to oxidative stress predisposition. These findings contribute to a sparse literature regarding the genetic underpinnings of BD-associated white matter anomalies, and reveal white matter tracts liable to oxidative stress-related damage. Future longitudinal studies with larger sample sizes are warranted to examine the temporal association between antioxidative defense genes and white matter anomalies early in the course of illness. Ultimately, this line of research has the potential to inform the integration of oxidative biology into the monitoring and treatment of early-onset BD.

## Supplementary information

Supplementary_Tables
