# Supplementary material for: Differential association of antioxidative defense genes with white matter integrity in youth bipolar disorder
Source: Transl Psychiatry. 2022 Dec 7;12:504. doi: 10.1038/s41398-022-02261-w (PMC9729619; doi:10.1038/s41398-022-02261-w)
Supplement: Supplementary file 1 — Supplementary_Tables [file 41398_2022_2261_MOESM1_ESM.docx]

**Supplementary Tables:**

**Supplementary Table 1:** *SOD2* rs4880 Whole Brain Gene Main Effect Post-hoc Analyses.

| Significant FA Clusters | Estimated Marginal Mean | | | Post-hoc (F; *p* ) | | |
| --- | --- | --- | --- | --- | --- | --- |
|  | AA | AG | GG | AA/AG | AG/GG | AA/GG |
| 1 | 0.445 | 0.450 | 0.488 | 0.23; 0.63 | 15.65; **<0.001*** | 14.61; **<0.001*** |
| 2 | 0.585 | 0.531 | 0.577 | 14.55; **<0.001*** | 14.73; **<0.001*** | 0.03; 0.86 |
| Note: FA = fractional anisotropy. Significant results are Bolded. *=Finding remains significant after correction for multiple comparisons (*p*<0.016). | | | | | | |

**Supplementary Table 2:** *SOD2* rs4880 Whole Brain Interaction Effect Post-hoc Analyses.

| Clusters | | Estimated marginal mean | | | | | | Post-hoc (F; *p*) | | | | | | | | |
| --- | --- | --- | --- | --- | --- | --- | --- | --- | --- | --- | --- | --- | --- | --- | --- | --- |
|  |  | BD_AA_ | BD_AG_ | BD_GG_ | HC_AA_ | HC_AG_ | HC_GG_ | BD_AA_/  BD_AG_ | BD_AG_/  BD_GG_ | BD_AA_/  BD_GG_ | HC_AA_/  HC_AG_ | HC_AG_/  HC_GG_ | HC _AA_/  HC _GG_ | BD_AA_/  HC_AA_ | BD_AG_/  HC_AG_ | BD_GG_/  HC_GG_ |
| FA | 1 | 0.683 | 0.671 | 0.649 | 0.666 | 0.657 | 0.714 | 0.84; 0.36 | 4.05; **0.047** | 6.35; **0.013** | 0.45; 0.50 | 18.65; **<0.001*** | 10.44; **0.002*** | 1.39; 0.24 | 1.71; 0.19 | 23.41; **<0.001*** |
|  | 2 | 0.482 | 0.465 | 0.465 | 0.453 | 0.482 | 0.522 | 2.23; 0.14 | 0.001; 0.97 | 2.17; 0.14 | 6.78; **0.01** | 12.73; **<0.001*** | 29.23; **<0.001*** | 5.08; **0.03** | 3.04; 0.08 | 25.39; **<0.001*** |
| RD | 1 | 0.464 | 0.481 | 0.495 | 0.499 | 0.481 | 0.441 | 1.08; 0.30 | 1.02; 0.31 | 3.33; 0.07 | 1.24; 0.27 | 5.64;  **0.02** | 9.39; **0.003*** | 3.35; 0.07 | 0.002; 0.96 | 10.41; **0.002*** |
|  | 2 | 0.349 | 0.351 | 0.386 | 0.360 | 0.373 | 0.321 | 0.01; 0.91 | 8.71; **0.004*** | 6.28; **0.01** | 0.83; 0.36 | 12.41; **<0.001*** | 5.39;  **0.02** | 0.45; 0.50 | 3.37; 0.070 | 19.07; **<0.001*** |
|  | 3 | 0.477 | 0.491 | 0.497 | 0.497 | 0.491 | 0.452 | 1.80; 0.18 | 0.63; 0.43 | 3.77; 0.06 | 0.36; 0.55 | 14.37; **<0.001*** | 14.95; **<0.001*** | 2.83; 0.10 | <0.01;  0.99 | 19.46; **<0.001*** |
|  | 4 | 0.489 | 0.497 | 0.505 | 0.503 | 0.498 | 0.454 | 0.75; 0.39 | 0.97; 0.33 | 2.69; 0.10 | 0.27; 0.60 | 18.83; **<0.001*** | 18.34; **<0.001*** | 1.69; 0.20 | 0.02; 0.89 | 25.07; **<0.001*** |
| BD = Bipolar disorder; HC = Healthy control; FA = fractional anisotropy; RD = radial diffusivity. Note: the estimated marginal mean for RD are scaled by 10^3^. Significant results are Bolded. *=Finding remains significant after correction for multiple comparisons (*p*<0.005). | | | | | | | | | | | | | | | | |
